# Clinical, economical and safety impact of ferric carboxymaltose use in Patient Blood Management programme in Portuguese National Health Service hospitals

**DOI:** 10.1038/s41598-022-21929-3

**Published:** 2022-11-11

**Authors:** Joana Lucas, Eduardo Costa, Ana Subtil, Rita Sequeira, Adalberto Campos Fernandes, António Robalo Nunes, Paulo Sousa

**Affiliations:** 1 Hospital Dr. Nélio Mendonça, Funchal, Portugal; 2 Nova School of Business and Economics, Lisbon, Portugal; 3 NOVA National School of Public Health, NOVA University Lisbon, Lisbon, Portugal; 4 NOVA National School of Public Health, Comprehensive Health Research Center (CHRC), NOVA University Lisbon, Lisbon, Portugal; 5 CEMAT, Instituto Superior Técnico, Universidade de Lisboa, Lisbon, Portugal; 6 Present Address: NOVA National School of Public Health, Public Health Research Centre, CISP, NOVA University Lisbon, Lisbon, Portugal; 7 Hospital das Forças Armadas, Lisbon, Portugal; 8 Universidade NOVA de Lisboa–NOVA Medical School, Lisbon, Portugal

**Keywords:** Health care, Anaemia, Outcomes research

## Abstract

Ferric carboxymaltose (FCM) can be used in Patient Blood Management (PBM) to promote the optimization of preoperative haemoglobin (Hb), which aims to minimise the use of allogeneic blood components and improve clinical outcomes, with better cost-effectiveness. This was an observational study conducted in a retrospective and multicentre cohort with adults from elective orthopaedic, cardiac and colorectal surgeries, treated according to local standards of PBM with allogeneic blood product transfusions (ABTs) on demand and with FCM to correct iron deficiency with or without anaemia. In this work, only the first pillar of the PBM model issue by Directorate-General for Health (DGS) was evaluated, which involves optimising Hb in the preoperative period with iron treatment if it’s necessary/indicated. Before the implementation of PBM in Portugal, most patients did not undergo preoperative laboratory evaluation with blood count and iron kinetics. Therefore, the existence of Iron Deficiency Anaemia (IDA) or Iron Deficiency (ID) without anaemia was not early detected, and there was no possibility of treating these patients with iron in order to optimise their Hb and/or iron stores. Those patients ended up being treated with ABTs on demand. A total of 405 patients from seven hospitals were included; 108 (26.7%) underwent FCM preoperatively and 197 (48.6%) were transfused with ABTs on demand. In the FCM preoperative cohort, there was an increase in patients with normal preoperative Hb, from 14.4 to 45.7%, before and after FCM, respectively, a decrease from 31.7 to 9.6% in moderate anaemia and no cases of severe anaemia after FCM administration, while 7.7% of patients were severely anaemic before FCM treatment. There were significant differences (p < 0.001) before and after correction of preoperative anaemia and/or iron deficiency with FCM in Hb, serum ferritin and transferrin saturation rate (TS). In the ABT group, there were significant differences between pre- and postoperative Hb levels (p < 0.001). Hb values tended to decrease, with 44.1% of patients moving from mild anaemia before transfusion to moderate anaemia in the postoperative period. Concerning the length of hospital stay, the group administered with ABTs had a longer hospital stay (p < 0.001). Regarding the clinical outcomes of nosocomial infection and mortality, there was no evidence that the rate of infection or mortality differed in each group (p = 0.075 and p = 0.243, respectively). However, there were fewer nosocomial infections in the FCM group (11.9% versus 21.2%) and mortality was higher in the transfusion group (21.2% versus 4.2%). Economic analysis showed that FCM could reduce allogenic blood products consumption and the associated costs. The economic impact of using FCM was around 19%. The preoperative Hb value improved when FMC was used. Patients who received ABTs appeared to have a longer hospital stay. The FCM group reported fewer infections during hospitalisation. The economic results showed savings of around €1000 for each patient with FCM administration. The use of FCM as part of the PBM program had a positive impact on patients’ outcomes and on economic results. However, it will be essential to perform studies with a larger sample to obtain more robust and specific results.

## Introduction

In order to minimise the use of blood products, as well as reduce transfusion-associated risks, strategies to optimise the use of allogeneic blood have been developed. Patient Blood Management (PBM) is a multidisciplinary approach that optimises own red cell mass, minimising blood loss and harnessing and optimising the patient-specific anaemia reserve^1,2^. Several studies have revealed that PBM reduces the number of blood components transfusions (ABTs) and decreases morbidity and mortality, with better clinical results and lower hospital costs^1,3–9^.

About 30% of surgical patients have preoperative anaemia and this number can reach 75% in those with colorectal pathology^10–13^. PBM can be used in the preoperative, intraoperative and postoperative period of several elective surgeries^2,4,14^ reducing the requirement for ABTs in cardiac or orthopaedic patient surgeries by up to about 55%^15^. According to the results of a systematic review and published meta-analysis, PBM reduced the overall mortality rate by 11%, with a decrease of 27% in orthopaedic surgery. There was also a reduction of 20% in complications as well as a reduction of 0.45 days in hospital length of stay^15^.

A Portuguese report from 2017 regarding PBM estimated a reduction of 51.2% in the number of ABTs per year, an 8.4% reduction in hospital admissions and a 37.3% reduction in readmissions^16^. At the economic level, the report indicated savings of approximately 70 million euros in relation to hospitalizations and 18 million euros in costs of blood products when implementing PBM^16^.

Early detection, evaluation and treatment of anaemia have a direct impact on surgical prognosis^4,8,9,17–22^. The global prevalence of anaemia ranges from 22.8 to 32.9%, and Iron Deficiency Anaemia (IDA) is the most common aetiology.

The Directorate-General for Health (DGS) has published a PBM model for general use in Portuguese hospitals. These guidelines at the preoperative phase should include detection and treatment of anaemia and iron deficiency, including treating underlying causes and optimising haemoglobin (Hb). According to this model, all the patients undergoing surgical procedures with expectation of bleeding, should have a laboratory evaluation with blood count and iron kinetics^23^.

A Hb value below 13 g/dL in men and below 12 g/dL in women is considered anaemia. Anaemia can be multifactorial, especially in the elderly or in those with chronic illness, kidney failure, nutritional deficiencies or malabsorption. In an adult patient with anaemia, a ferritin level < 15 μg/L is diagnostic of iron deficiency (ID) and levels between 15 and 30 μg/L are highly suggestive. However, ferritin is elevated in inflammation, infection, liver disease and malignancy, which can result in erroneously elevated ferritin levels in patients where iron deficiency and systemic disease coexist. In the elderly or in patients with inflammation, iron deficiency may still be present with ferritin values up to 60–100 μg/L. Therefore, in cases where ferritin < 100 μg/L, iron therapy should be considered if a loss of about 3 g/dL of Hb is anticipated^23^.

In the preoperative period, the first line therapy of IDA or ID should be oral iron. Its efficacy must be evaluated at least 1 month later. If anaemia has not been corrected, or if there is a contraindication or intolerance to oral iron, or if more rapid replenishment of iron stores is necessary, the use of intravenous iron is recommended^23^.

Ferric carboxymaltose (FCM) has several benefits in the management of iron deficiency in the surgical context^24–28^. Compared to other parenteral iron formulations, FCM can be administered in a single session and provides better results in the treatment of IDA^29^. In comparison with oral iron, it is able to quickly replace iron stores with a low rate of side effects^29^. In a meta-analysis that evaluated the use of FCM in the perioperative period to improve haematological parameters and reduce the number of postoperative transfusions, Hb, serum ferritin and the transferrin saturation rate (TS) were higher in those who used FCM compared to oral iron or placebo^27^. FCM has a low rate of side effects, even in comparison with other intravenous formulations^30,31^. It presented an adverse reaction rate of 12% in relation to sucroferric oxyhydroxide, iron dextran and iron isomaltoside, which presented rates of 15.3%, 12% and 17%, respectively^30^. Moderate to severe hypophosphataemia is a common effect with intravenous iron, usually without clinical symptoms, the incidence of which is variable^32^.

A study performed at Zurich University Hospital evaluated transfusion data before and after implementation of PBM and the sustainability of this regimen. The greatest impact was seen in the number of ABTs, with a reduction of 35%, in comparison to the years 2012 and 2017, and a consequent economic gain of 12,440,000 euros^9^. Another economic study that considered iron supplementation and the use of tranexamic acid concluded that the need for transfusion declined from 30 to 18%, the average transfusion cost declined from 68 to 32 euros and the average hospital stay was less than 0.45 days, resulting in a saving of 114 euros per patient^33^.

This work aimed to evaluate the impact of haemoglobin optimisation with correction of preoperative IDA with FCM, as part of the implementation of PBM, in patients proposed for elective orthopaedic (hip or knee arthroplasty), cardiac or colorectal surgery, in Portuguese hospitals.

## Materials and methods

This was an observational study conducted in a retrospective and multicentre cohort design with data collection from medical and administrative records (Table 1). The study took place in a group of Portuguese National Health Service hospitals, which began implementation of the PBM programme developed by DGS at the end of the first half of 2019^23^. Consecutive male and female patients, over the age of 18, with IDA or ID, who underwent elective orthopaedic (hip or knee arthroplasty), cardiac and colorectal surgeries during the years 2017, 2018, 2019 and 2020, were selected for inclusion in the study. Pregnant women were not included. IDA was considered in male patients with preoperative Hb lower than 13 g/dL or lower than 12 g/dL for females^26^. In the presence of normal Hb values, patients with serum ferritin levels below 100 μg/L were considered as ID and were also included in the study^26^, since iron therapy should be considered in this case if a loss of about 3 g/dL of Hb is expected. The types of surgeries included in this study are related to high rates of bleeding loss. Each participating hospital performed at least one of the three types of surgery.

**Table 1:** Results of descriptive statistics.

Variables	Descriptive statistics
(N = 405)	
**Age (in years)**	
Mean (SD)	72.3 (11.2)
Median (IQR)	74.0 (67.0–80.5)
Min–max	21–98
**Gender**	n (%)
Male	209 (51.6%)
Female	196 (48.4%)
**Type of surgery**	n (%)
Cardiac	174 (43.0%)
Colorectal	139 (34.3%)
Orthopedic	92 (22.7%)
**Length of hospital stay (days) (N = 374)**	
Mean (SD)	13.3 (21.0)
Median (IQR)	8.0 (6.0–13.3)
Min–max	0–341
**Infections during hospitalization**	n (%)
Yes	64 (15.8%)
No	341 (84.2%)
**Hospital mortality**	n (%)
Yes	21 (5.2%)
No	365 (90.1%)
**Mortality within 30 days of hospital discharge**	n (%)
Yes	3 (0.7%)
No	401 (99.3%)
**Preoperative FCM**	n (%)
Yes	108 (26.7%)
No	297 (73.3%)
**Date of first FCM dose (days before surgery) (N = 108)**	
Mean (SD)	57.6 (55.2)
Median (IQR)	47.5 (14.0–77.5)
Min–max	0–251
**FCM adverse reactions**	n (%)
Yes	0 (0%)
No	65 (100%)
**Blood components administration**	n (%)
Yes	197 (48.6%)
No	208 (51.4%)

In the post-PBM implementation, the use of FCM preoperatively to correct iron stores was the most common therapeutic method. While, in the pre-PBM implementation, most patients did not undergo laboratory evaluation to assess the existence of IDA or ID, so most commonly only those with severe anaemia were treated with ABTs on-demand. We obtained data from both before and after PBM implementation which allowed us to collect data regarding the two therapeutic methods.

The primary outcome of this study was the Hb values after the treatment with FCM in the preoperative period. The secondary outcomes were: The ferritin and TS levels after the treatment with FCM in the preoperative period; the Hb values after the ABTs; the rate of nosocomial infections, length of stay and mortality; the direct and indirect costs of the FCM and ABTs use.

To carry out this research we defined two groups, taking into account all operative periods (pre, intra and postoperative): the group that only received FCM (pre and/or postoperatively) and the group that received at least one ABT (pre, intra and/or postoperative). In this group, all types of transfusion (red blood cells, fresh frozen plasma, and platelet concentrates) were included, since these patients did not undergo PBM strategies to optimise Hb before surgery and were therefore more likely to have complications, and consequently greater blood consumption. Although the two groups are not directly comparable, the analysis of the ABT group aimed to assess additional data, such as variations in Hb level, as well as hospital costs, that might indirectly reinforce or not the relevance of FCM therapy.

Data was processed using Excel 2017 software and analysed using IBM SPSS Statistics (Statistical Package for the Social Science) version 27.

Descriptive analysis was carried out, as well as inferential analysis, in which diverse statistical tests were applied (Wilcoxon signed rank test, chi-square test, Fisher's exact test, and Mann–Whitney–Wilcoxon test), performed at the 0.05 level of significance.

The Wilcoxon signed rank test was applied to compare Hb, ferritin and TS values before and after correction of preoperative IDA or ID with FCM. The same test was applied to compare pre and postoperative Hb levels for patients who received ABTs on demand. Since we wanted to compare repeated measurements of Hb, ferritin and TS levels obtained from the same sample, but these values were measured on an ordinal scale, we chose to apply the Wilcoxon signed rank test, a non-parametric alternative to the t-test for paired samples, only suitable for data measured on a ratio or interval scale.

The chi-square test was applied to assess whether the infection rate was different between patients who received FCM only and those who received ABTs only, because this test is suitable for assessing the differences in the distribution of a categorical variable between independent groups. In the case of hospital mortality, we chose to apply the Fisher's exact test instead of the chi-square test, since more than 20% of the cells in the contingency table had an expected frequency of less than 5.

### Ethics approval and consent to participate

The authors declare that they complied with ethical principles according to the Declaration of Helsinki. The study was a retrospective study based on anonymous and aggregate data. However, the study was approved by the boards of all participants hospitals and was also approved by two ethical committees namely Hospital Professor Doutor Fernando da Fonseca’s ethical committee (nº 47/2020) and Centro Hospital Lisboa Central’s ethical committee (Nº 1160).

Since this was a retrospective study based on anonymous and aggregate data, the informed consent was not required by the Hospital Professor Doutor Fernando da Fonseca’s ethical committee and the Centro Hospital Lisboa Central’s ethical committee.

## Results

Data collection, carried out from December 2020 to September 2021, identified 633 patients from seven Portuguese hospitals. Out of these, 228 were excluded due to filing errors, insufficient information or because they did not fulfil the inclusion criteria. Thus, we present the analysis of 405 patients, of which 108 (26.7%) underwent FCM preoperatively, 197 (48.6%) were administered ABTs on demand, and 124 (30.6%) did not undergo any of these therapies, but they also met the inclusion criteria. The majority were male (51.6%) and the age range was between 21 and 98 years with a mean (SD) of 72.3 (11.2) years (Table 1).

In this study, 54.3% of the patients had mild preoperative IDA, 31.4% had moderate preoperative IDA and 4.9% had severe preoperative IDA (Table 2). In terms of iron kinetics, Table 3 and Table 4 display the sample characteristics regarding serum ferritin and TS levels.

The 108 patients (26.7%) in the FCM preoperative cohort received a dose of at least 500 mg and/or 1000 mg FCM preoperatively on a mean (SD) of 57.6 (55.2) days before surgery. When FCM was administered 4 to 6 weeks before surgery, the Hb value improved. The percentage of patients with normal Hb values increased from 14.4% before FCM treatment to 45.7% afterwards. As for moderate IDA, there was a decrease in cases from 31.7 to 9.6% after FCM treatment, and there were no cases of severe IDA in patients who received FCM, while 7.7% were affected before this therapy. Regarding the values of serum ferritin and TS, the results were similar with an increase in the percentage of patients within the normal range; for serum ferritin the increase was from 16.4 to 50.8% and for TS the increase was from 9 to 41.4%, although both parameters presented a high percentage of missing data. Inferential statistics using the Wilcoxon test for paired samples provided evidence that there were significant differences before and after correction of preoperative IDA or ID with FCM (p < 0.001). In all analyses, the differences between the paired data indicated that Hb, ferritin and TS levels improved after the administration of FCM.

**Table 2:** Number of patients according the different types of anemia at preoperative period, including pre and post transfusion moments, and after the treatment of anemia/ iron deficiency correction.

Sample size (N = 405)	Phases of surgery			
	Preoperative (N = 405)	Preoperative pre-transfusion (N = 321)	Preoperative post-transfusion (N = 322)	Preoperative after anemia and/or iron deficiency correction (N = 214)
	N (%)	N (%)	N (%)	N (%)
Normal	36 (8.9%)	0 (0.0%)	0 (0.0%)	56 (17.1%)
Mild anemia	220 (54.3%)	1 (0.3%)	1 (0.3%)	55 (16.7%)
Moderate anemia	127 (31.4%)	8 (2.5%)	17 (5.3%)	15 (4.5%)
Severe anemia	20 (4.9%)	15 (4.7%)	3 (0.9%)	0 (0.0%)
Missing analysis	2 (0.5%)	297 (92.5%)	301 (93.5%)	204 (61.8%)

**Table 3:** Number of patients according the different values of serum ferritine at preoperative period and after the treatment of anaemia/ iron deficiency correction.

Sample size (N = 405)		Phases of surgery	
		Preoperative (N = 385)	Preoperative after anemia and/or iron deficiency correction (N = 337)
		N (%)	N (%)
Ferritine > 100 µg/L (normal)		45 (11.7%)	33 (9.8%)
Ferritine 30-100 µg /L		59 (15.3%)	5 (1.5%)
Ferritine 15–30 µg /L		33 (8.6%)	1 (0.3%)
Ferritine < 15 µg/L		33 (8.6%)	0 (0.0%)
Missing analysis		215 (55.8%)	298 (88.4%)

**Table 4:** Number of patients according the different values of transferrin saturation at preoperative period and after the treatment of anaemia/ iron deficiency correction.

Sample size (N = 405)	Phases of surgery	
	Preoperative (N = 382)	Preoperative after anemia and/or iron deficiency correction (N = 329)
	N (%)	N (%)
TS 20–45% (normal)	45 (11.8%)	27 (8.2%)
TS < 20%	112 (29.3%)	10 (3.0%)
Missing analysis	225 (58.9%)	292 (88.8%)

Out of the total sample, 48.6% received ABTs on demand. In this group, there were significant differences (p < 0.001) between pre and postoperative Hb levels. Hb values tended to decrease, with 44.1% of the patients moving from mild anaemia before transfusion to moderate anaemia in the postoperative period. Differences in ferritin and TS values could not be analysed statistically due to the small number of observations.

In terms of length of hospital stay, there was a significant difference between the group who received FCM and the ABTs group (p < 0.001), according to the Mann–Whitney-Wilcoxon test. The group administered ABTs was assigned higher orders in the test, suggesting that, in general, it presented longer hospital stays. This is consistent with descriptive statistics that showed that the median stay for the FCM group was 6 days and for the other group it was 10 days. The majority of patients who were treated with FCM (82.1%) had a hospital stay of 10 days or less and only 9.0% stayed between 10 and 20 days, and 8.9% of 20 days or more. In the patients treated with ABTs, 50.7% had a hospital stay of 10 days or less, 30.8% between 10 and 20 days and 18.5% of 20 days or more Table 5). R﻿egarding clinical outcomes such as nosocomial infections, according to the chi-square test there was no evidence (p = 0.075) that the rate of infections differed between the two groups. However, in the FCM group there were fewer nosocomial infections (11.9% versus 21.2%). In the analysis of hospital mortality, according to the Fisher's exact test, there was no evidence (p > 0.999) that mortality differed between the two groups (Table 5).

**Table 5:** Comparative analysis between FCM and ABT group concerning the length of hospital stay, infections during hospitalization and hospital mortality.

Variables	FCM [N = 84]	ABT [N = 156]	p-value
**Length of hospital stay (days)**	[N = 66]	[N = 146]	
Median (IQR)	6.0 (5.0–8.0)	10.0 (7.0–17.3)	< 0.001^a^
**Infections during hospitalization**			
Yes n (%)	10/84 (11.9%)	33/156 (21.2%)	0.075^b^
No	74/84 (88.1%)	123/156 (78.8%)	
**Hospital mortality**			
Yes n (%)	3/71 (4.2%)	7/155 (21.2%)	> 0.999^c^
No	68/71 (95.8%)	148/155 (78.8%)	

^a^Mann–Whitney-Wilcoxon test applied; ^b^Chi-square test applied; ^c^Fisher’s exact test applied.

Concerning the economic analysis, the average cost of FCM per patient was €267. This was estimated based on the average consumption of FCM per patient (in milligrams), and the pharmaceutical cost of FCM. Regarding the consumption of ABTs, when comparing the group that only used FCM with the group that was only treated with ABTs, significant differences were identified. Namely, the consumption of blood products by the FCM group was lower. Thus, the transfusion costs for the FCM group were over 70% lower than the group treated only with ABTs (142€ vs 582€). Considering that FCM and ABTs are the main determinants of direct costs in this study, the FCM group incurred lower direct costs than the ABT group—the average saving per patient was more than 170€ (Tables 6 and 7).

**Table 6:** Direct cost of FCM.

	Amount (mg)	Price (€)	Cost
FCM	1543	0.17	267.18€
Blood components	0.0	0	− €
Variation	− 1543		− 267.18€

**Table 7:** Direct cost of ABTs.

	Red blood cells	Platelet transfusion	Frozen plasma	Total
FCM	95.52 €	43.04 €	3.69 €	142.26 €
Blood components	301.24€	243.37€	37.82€	582.43€
Variation	205.73 €	200.32 €	34.12 €	440.17 €

We also considered changes in length of hospital stay, which is considered to be an indirect cost. The length of stay for patients treated with FCM was, on average, 2.8 days shorter than for patients who received ABTs. Based on hospital tariffs, this reduction implies an estimated saving of €864 per patient (Table 8). Hence, the joint analysis of direct (FCM and ABTs) and indirect costs (length of stay) suggests that patients treated with FCM incurred lower costs than patients treated only with ABTs. The cost difference was estimated at €1037 per patient, representing 19% of the total cost (Table 9).

**Table 8:** Costs of hospital stay (€/patient).

	Hospital stay (mean)	Price (€)	Cost
FCM	13.1	304.27 €	3979.83 €
Blood components	15.9	304.27 €	4843.96 €
Variation	2.8		864.12 €

**Table 9:** Direct and Indirect costs (€/patient).

Costs	FCM group	ABT group	Variation
Hospital stay	3979.83 €	4843.96 €	864.12 €
FCM	267.18 €	– €	− 267.18€
Blood components	142.26 €	582.43 €	440.17 €
Total	4389.27 €	5426.39 €	1037.12 €

## Discussion

In this study, the results demonstrated a significant difference between the Hb values and serum markers of iron (ferritin and TS values) before and after correction of preoperative IDA or ID (p < 0.001) in patients who used FCM in the preoperative period. After treatment with FCM, the number of patients with normal Hb values and mild IDA increased and the number of patients with moderate IDA decreased, and there were no cases of severe IDA. Thus, there was a clear impact of FCM on optimising Hb values in the preoperative period, which helped improve the IDA profile of these patients^34^.

This study has some limitations, mainly due to the sample size, which precluded the use of certain statistical tests to clarify the association between some of the variables, and an analysis according to different subgroups such as the type of surgery, and FCM dosage for example. Another important limitation was the large amount of missing laboratory data, such as those related to post-transfusion Hb assessment, as well as the high percentage of cases without serum iron markers. This lack of information conditioned the analysis of the ABT group, where the absence of these data predominated, and it was not possible to perform statistical tests in the same way as for the FCM group. All these missing data hampered more robust statistical studies in the ABT group.

This study was carried out in the context of the PBM program issued by the DGS^23^, it should be noted that it only focuses on the first pillar of the PBM, which concerns the optimization of preoperative Hb with iron treatment. Therefore, other PBM strategies that are equally important to reduce allogeneic blood consumption and improve patient outcomes^15,17^, such as strategies to reduce blood loss during the surgery and the use of hemostatic agents, were not contemplated in this work.

The economic analysis had a significant set of limitations and was specific to the context of the study carried out. The cost estimate focused only on the direct costs (FCM utilisation and ABTs) and indirect costs of hospitalisation. It did not consider other costs and benefits that may exist, such as a reduction in the rate of complications, or changes in the consumption of other therapies. Moreover, the results rely on the assumption that the two groups of patients were comparable. However, if this assumption was violated, then patient selection between groups may have introduced a bias in the estimates.

These results are consistent with the available evidence, such as the work published in 2021 by Peel et al., which proved that the use of high doses of intravenous iron (more than 600 mg) was associated with a significant increase in preoperative Hb in the context of heart surgery^35^. According to Morais et al., Hb optimisation, in this same surgical context, is extremely clinically relevant, as a 1 g/dL drop in Hb was associated with a higher need for blood transfusions, longer hospital stays, and a higher mortality rate^27^. Due to the small size of the sample, it was not possible to establish an association between the use of FCM and a possible reduction in the number of ABTs performed in this group of patients.

In patients with ABTs, the Hb levels were not optimised before and after the transfusions. Although the number of patients with severe IDA was small at the time of hospital discharge, the percentage with moderate IDA was higher compared to the preoperative period. This finding can be explained by the fact that patients with severe IDA in the preoperative period and who received red blood cells transfusions consequently had an increase in Hb level, which reduced the severity of IDA to moderate^36^. There was also a reduction in cases of mild IDA in the postoperative compared to the preoperative period, which can be explained by intraoperative and postoperative blood loss, coagulopathy, high number of phlebotomy, postoperative reduced erythropoiesis due to surgery-associated inflammation, hemodilution from excessive perioperative fluids, other nutritional deficiencies, pharmacological interactions and low transfusion efficiency, all of which could have decreased haemoglobin levels and worsened some patients profile.

The available literature on the use of FCM and the reduction of hospital stay presents inconsistent results, without robust evidence to corroborate a clear beneficial relationship between these two variables^34^. In the current study, the percentage of patients with a hospital stay of 10 days or less was higher in the FCM group (82.1% in the FCM group versus 50.7% in the ABT group). Although these results may indicate a benefit of FCM in reducing hospital stay, they may be associated with other variables that were not taken into account, such as the clinical context of each therapy. It will be important to carry out other studies that include variables such as other therapies, the existence of clinical complications and ICU admissions, in order to confirm the existence and the real extent of the association between the use of FCM and the length of hospital stay. A recent systematic review showed that there was a 9% reduction in nosocomial infections with PBM implementation^15^. Data from the present study show that 15.8% of patients had infections during hospitalisation. In this sample, according to the chi-square test and Fisher’s exact test, there was no statistical evidence of differences in the number of infections and mortality between the two groups of patients. However, the group with ABTs had a higher rate of infections (21.4%), while the FCM group seemed to have a lower rate (11.9%). Further studies are necessary to take into account possible variables such as the age and general condition of the patient, type of surgery, co-morbidities and possible therapies performed, to establish and confirm or invalidate these statistical relationships.

Finally, no side effects from the use of FCM and the ABTs were reported. This may be related to factors such as the sample size or the underreporting of adverse events, especially concerning blood transfusions, where the risk of infection and other adverse reactions is well known, and the notification of these events is not always performed^32,35^. In the case of FCM, the low reported incidence of side effects was expected based on previous studies^29,30,31^.

From a health system perspective, it is relevant to estimate the direct costs involved in reducing the consumption of blood components, as well as the costs associated with FCM. The goal of PBM is to minimise the consumption and use of blood products^16,23^. This study showed that FCM could reduce this consumption and the associated costs. In fact, there were significant differences between the two groups: on average there was a 70% decrease in the FCM group. Although there was an increase in costs due to FCM, there was also a significant reduction in transfusion costs and hospital length of stay. Overall, the total cost per patient in the FCM group was 19% lower than that for patients treated only with blood transfusions.

In this study, 124 patients out of the total study population had IDA and/or ID and did not receive preoperative FCM or ABTs. This may mean that, although participating hospitals already have PBM programmes, they may not be systematically applied in clinical practice. Although PBM strategies are well-founded and defined, it is necessary to adapt them to the reality of each hospital.

## Conclusions

This study allowed us to conclude that FCM has a positive impact on optimising Hb and iron stores, promoting the correction or improvement of preoperative iron deficiency with or without IDA in patients undergoing elective orthopaedic, cardiac and colorectal surgeries.

Regarding post-surgical morbidity factors, the data revealed an association between FCM use and length of hospital stay. The group that received FCM had fewer days of hospitalisation when compared to the patients who received ABTs, as well as a lower incidence of infections.

Concerning the economic results, patients treated with FCM incurred lower costs than patients treated only with ABTs. The cost difference was estimated at €1037 per patient, representing 19% of the total cost. This cost reduction was positively affected by the decrease in consumption of blood products and the length of hospital stay, but negatively affected by the costs of FCM.

Overall, this study demonstrates a potential clinical and economic benefit from the use of FCM. However, it will be essential to perform studies with a larger sample to obtain more robust and specific results.

## Data Availability

The datasets used and/or analysed during the current study are available from the corresponding author on reasonable request.
